# The role of magnetic sphincter augmentation in the gastroesophageal reflux disease treatment pathway: the gastroenterology perspective

**DOI:** 10.1093/dote/doad005

**Published:** 2023-02-10

**Authors:** Amit Patel, C Prakash Gyawali

**Affiliations:** Division of Gastroenterology, Duke University School of Medicine and the Durham VA Medical Center, Durham, NC, USA; Division of Gastroenterology, Washington University School of Medicine, Saint Louis, MO, USA

**Keywords:** ambulatory reflux monitoring, gastroesophageal reflux disease, high-resolution manometry, magnetic sphincter augmentation, refractory GERD, regurgitation

## Abstract

Magnetic sphincter augmentation (MSA) is a surgical intervention for well-characterized gastroesophageal reflux disease (GERD), where the esophagogastric junction barrier is augmented using a bracelet of magnetized titanium beads. MSA could be an attractive option for patients with documented GERD who wish to avoid long-term pharmacologic therapy or whose symptoms are not adequately managed with lifestyle modifications and pharmacologic therapy. The ‘ideal’ MSA patient is one with prominent regurgitation, without dysphagia or esophageal motor dysfunction, with objective evidence of GERD on upper endoscopy and/or ambulatory reflux monitoring. Appropriate candidates with significant hiatus hernia may pursue MSA with concomitant hiatus hernia repair. The increasing adoption of MSA in the GERD treatment pathway reflects research that shows benefits in long-term outcomes and healthcare costs compared with other established therapies in appropriate clinical settings.

## INTRODUCTION

Gastroesophageal reflux disease (GERD) carries a high symptomatic and economic burden, with estimated prevalence of 9–28% in Western populations,^1^ and with an estimated >$9–10 billion/year of direct costs in the US alone, primarily related to proton pump inhibitor (PPI) use.^2^ In recent years, concerns about long-term PPI therapy have been widely publicized, despite the lack of objective hard evidence linking most of these purported adverse effects directly to PPIs.^3^ Consequently, there is high interest among the medical community as well as among GERD patients for potential alternatives to long-term PPI use. While laparoscopic antireflux surgery (ARS) remains the surgical standard for GERD, uncertain durability and undesirable new symptoms (such as bloating, delayed gastric emptying, and dysphagia) need to be considered in management decisions. Magnetic sphincter augmentation (MSA) is a minimally invasive alternative to PPI use that has become an attractive option in patients who have well-characterized GERD and do not favor either long-term PPI therapy or laparoscopic ARS. MSA consists of the laparoscopic implantation of a bracelet of magnetized titanium beads at the esophagogastric junction (EGJ) to augment the mechanical barrier against reflux.^4^ This article will discuss the gastroenterologist perspective to the indications, contraindications, and consequences of MSA use in GERD management.

## CLINICAL EVALUATION OF THE SYMPTOMATIC PATIENT

In typical GERD presentations of heartburn, chest pain and/or regurgitation and without alarm symptoms, a pragmatic initial approach consists of an empiric PPI trial.^5–7^ If symptoms improve, medication is continued at the lowest dose that improves symptoms, with the option of weaning further to histamine 2 receptor antagonists.^8^ If symptoms do not improve, if presentation is atypical (isolated hoarseness, cough, globus, or wheezing), and/or if alarm symptoms exist, further diagnostic evaluation is indicated to establish or rule out the presence of GERD prior to escalation of management.^8^^,^  ^9^ This diagnostic work-up typically starts with an upper endoscopy, which may reveal conclusive evidence of reflux-induced mucosal damage (reflux esophagitis, peptic stricture, long segment Barrett’s esophagus), supportive evidence for GERD (hiatus hernia), or evidence for an alternate diagnosis (eosinophilic esophagitis, other mucosal processes, achalasia, rarely cancer).^7–9^

Further evaluation in endoscopy-negative (unproven) GERD may include ambulatory reflux monitoring performed off PPI therapy, where findings of elevated distal esophageal acid exposure time (AET, >6.0%), high numbers of reflux events (>80), and/or low distal mean nocturnal baseline impedance (<1500 ohms) may constitute stand-alone evidence for GERD.^9–11^ Ambulatory reflux monitoring can also demonstrate other adjunctive markers for GERD, including positive reflux–symptom association and low post-reflux swallow induced peristaltic wave index (PSPWI), which provide supportive evidence when primary metrics remain inconclusive for GERD, particularly when AET is 4–6%. In conjunction with a normal endoscopy, AET <4.0%—especially when demonstrated on multiple days on a prolonged wireless pH study—and numbers of reflux episodes <40 represent conclusive evidence against GERD, and grounds for discontinuation of PPI therapy.^12^^,^  ^13^ In addition, behavioral disorders, particularly rumination syndrome, should be carefully excluded, where cognitive and behavioral therapy and diaphragmatic breathing are recommended in place of escalation of antireflux therapy.^5^

In selected patients with confirmed or proven GERD, where symptoms and/or complications are inadequately managed with lifestyle modifications and pharmacologic therapies, endoscopic and surgical antireflux interventions are management considerations.^4^^,^  ^5^ Additionally, some patients with proven GERD may pursue invasive intervention as a means to avoid long-term PPI use. Consequently, the pursuit and selection of antireflux interventions should be based on shared decision-making between patients and providers, where the provider considers patient symptoms, EGJ morphology, esophageal motor function, and co-morbidities while explaining all available management options, and the patient makes an informed decision taking their overall management preference into consideration.^8^ One of the management options currently offered in such scenarios is MSA, which has the advantage of not altering foregut anatomy in contra-distinction from ARS and transoral incisionless fundoplication. Consequently, the physician stake-holders in clinical decision-making need to be well versed with the indications, contraindications, risks, and benefits of MSA.

## INDICATIONS—THE ‘IDEAL PATIENT’ FOR MSA

MSA was first introduced into clinical practice approximately a decade ago,^14^ and several long-term studies have now been published confirming efficacy.^15–18^ Post hoc evaluation of the pivotal studies that led to FDA approval of the device, as well as high-quality randomized trial data, have provided key characteristics that describe the phenotype of the ideal MSA patient. Inclusion into these studies have consisted primarily of proven GERD patients with prominent regurgitation symptoms in the absence of a large hiatus hernia, implying increased frequency of transient lower esophageal sphincter relaxation (TLESR) as the dominant GERD mechanism. Multicenter studies that randomized patients with persistent moderate to severe regurgitation despite once-daily PPI to either MSA or twice-daily PPI have demonstrated significantly higher rates of regurgitation relief with MSA at 6-month follow-up (89% vs. 10%)^19^ and at 12-month follow-up (96% vs. 19%).^18^ Additionally, post hoc analysis of data from these studies demonstrated that reduction of total numbers of reflux episodes on pH-impedance monitoring to <40 was associated with improved symptomatic outcomes after MSA, and that pathologic numbers of reflux episodes (>80) on testing prior to MSA predicted symptomatic improvement.^13^ Thus, the ideal patient in whom MSA has clear advantages over PPI has proven GERD with regurgitation-predominant symptoms, in the absence of rumination syndrome. This is an important phenotype for consideration of MSA, since regurgitation is not well-controlled with PPI therapy alone, and mechanical intervention has been documented to have higher value and efficacy in controlling GERD-related regurgitation.^4^

Despite regurgitation representing the primary symptomatic target of the pivotal MSA studies, there is ample evidence that heartburn in the context of proven GERD also improves with MSA. Moderate to severe heartburn decreased from 89% at baseline to 12% at 5 years in a well-characterized international cohort treated with MSA.^15^ Among randomized trial data of regurgitation-predominant proven GERD patients, heartburn symptoms also responded to MSA.^18^ In this setting, abnormal reflux monitoring confirming GERD, and PPI response demonstrating that control of reflux burden improves reflux-associated heartburn, are supportive for prediction of MSA efficacy. Consequently, heartburn-predominant PPI responders with proven GERD who want to move away from chronic medication therapy are potential candidates for MSA.

The pivotal studies that evaluated MSA excluded patients with high body mass index (BMI), large hiatus hernias, prior foregut surgery, and/or complications of GERD. The modern-day invasive options for patients with morbid obesity include Roux-en-Y gastric bypass (RYGB), which can reduce GERD symptoms, including regurgitation, by disconnecting the major portion of the acid-producing stomach from continuity with the esophagus.^4^ This provides not just reflux symptom control but also overall improvement in obesity-related co-morbidities and quality of life, albeit at the cost of a restrictive diet, post-bariatric complications, and nutritional consequences. Large hiatus hernias are associated with significant disruption of the EGJ barrier and typically warrant dedicated repair for symptom benefit; consequently, MSA alone may not be sufficient in this setting. Prior esophageal surgery, especially disrupted fundoplication, requires repair of the fundoplication rather than just augmentation of the LES high pressure zone. MSA continues to be evaluated in settings where the EGJ anatomy is not disrupted, such as prior myotomy or prior sleeve gastrectomy. Finally, GERD complications require independent management of the complication apart from approaches to reduce reflux. Therefore, it is reasonable to consider alternate management options in morbidly obese patients, and patients with GERD complications, large hiatus hernias, and prior surgery.

As post-MSA dysphagia is the most common reason for device removal,^14^^,^  ^20^ the presence of preoperative dysphagia or esophageal disorders that could contribute to postoperative dysphagia may not be ideal when MSA is being considered. In a retrospective review of 380 patients, the presence of preoperative dysphagia significantly predicted persistent dysphagia at >3 months after MSA on logistic regression (odds ratio 2.19).^21^ In the context of reflux symptoms, etiology of dysphagia may often be structural (strictures, concomitant eosinophilic esophagitis), which can be identified on endoscopy with biopsy, and perhaps a barium swallow.

Rarely, achalasia spectrum disorders can present with symptoms mimicking GERD. Esophageal high-resolution manometry (HRM) serves to exclude achalasia spectrum disorders prior to invasive reflux management and needs to be performed prior to MSA for similar reasons.^22^ Although high-quality data are limited on esophageal hypomotility disorders leading directly to dysphagia after an antireflux intervention, ineffective esophageal motility and absent contractility could potentially contribute to postoperative dysphagia in the setting of an obstructing antireflux intervention. Therefore, while not representing an absolute contraindication, caution should be encouraged when considering MSA in the context of significant esophageal hypomotility.

The multiple rapid swallow (MRS) maneuver during HRM can be particularly helpful in this setting, through assessment for contraction reserve, defined as augmentation of esophageal body contractile vigor following MRS compared with supine wet swallows.^23^ Based on data associating the absence of contraction reserve with late post-ARS dysphagia and esophageal motor dysfunction after ARS,^24^^,^  ^25^ the presence of contraction reserve on HRM may mitigate the risk of dysphagia after MSA. In one retrospective review of 68 patients who underwent MSA, proportions of patients with intact contraction reserve were higher among those without dysphagia (46% vs. 6%).^26^ Overall, MSA appears to increase mean LES resting pressures and integrated relaxation pressures, with compensatory increases in esophageal body contraction vigor, similar to that seen following the MRS maneuver.^27^ Thus, the presence of contraction reserve following MRS could predict augmentation of esophageal body peristalsis following MSA. Among patients without dysphagia after MSA, the upper limit of normal for intrabolus pressures on post-treatment HRM was approximately 30 mmHg across two different cohorts,^28^^,^  ^29^ suggesting that exceeding this threshold on pretreatment HRM may predict post-MSA dysphagia.

**Fig. 1:**
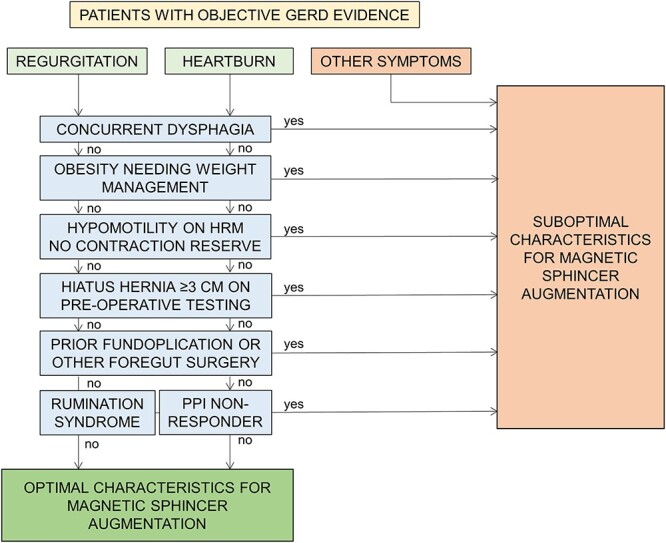
Clinical algorithm for selection of the ‘ideal’ patient for magnetic sphincter augmentation (MSA). A proposed algorithm for identification of the optimal patient for magnetic sphincter augmentation. Regurgitation predominant symptoms, without dysphagia, with no clinically relevant obesity, no significant hypomotility on high-resolution manometry (especially without contraction reserve on multiple rapid swallows), no hiatus hernia ≥3 cm in size and no prior foregut surgery are the ideal MSA patient characteristics. In case of heartburn, response to medical management is a further optimal criterion, since heartburn unresponsive to proton pump inhibitors could be related to esophageal hypersensitivity. Expanded criteria for magnetic sphincter augmentation continue to be studied (Table 1).

Therefore, the ideal patient for MSA has proven GERD (with endoscopic and/or ambulatory reflux monitoring evidence), without dysphagia or significant esophageal motor dysfunction apparent on HRM, with predominant regurgitation symptoms despite optimal medical management and without significant hiatus herniation (unless concomitant hernia repair is considered) (Fig. 1).^4^^,^  ^30^ Appropriate preoperative work-up therefore includes a careful history, upper endoscopy, esophageal HRM, ambulatory reflux monitoring, and/or barium imaging.

## EXPANDED INDICATIONS FOR MSA

There has been interest in the expansion of potential indications for MSA to include ongoing reflux in post-surgical patients, such as after sleeve gastrectomy, RYGB, or myotomy for achalasia (Table 1). Although sleeve gastrectomy may be effective for weight loss, it has been associated with de novo, persistent, and/or worsening GERD in the setting of decreased LES tone, disruption of the angle of His and/or sling fibers, decreased gastric emptying, and increased intragastric pressures. A retrospective analysis of 22 patients with persistent or de novo GERD after sleeve gastrectomy demonstrated significant post-MSA improvements in mean GERD-HRQL scores (44 to 17), with 82% able to come off anti-acid medications.^31^ Another cohort of 13 patients with MSA implantation performed for reflux after sleeve gastrectomy demonstrated mean GERD-HRQL score improvement from 47 to 12.^32^ In selected patients, MSA may therefore represent an alternative to conversion to RYGB for the management of GERD after sleeve gastrectomy.

**Table 1:** Expanded Indications for magnetic sphincter augmentation (MSA)

**Patients with proven gastroesophageal reflux disease**
Reflux following sleeve gastrectomy
Concurrent with repair of hiatus hernia of ≥3 cm
Persisting regurgitation following Roux-en-Y gastric bypass
Reflux following myotomy, including per-oral endoscopic myotomy (POEM)
Weakly acidic reflux with reflux-symptom association on pH-impedance monitoring
Reflux associated with Barrett’s esophagus, including following endoscopic intervention

While RYGB represents a more effective bariatric intervention for reflux than sleeve gastrectomy due to disconnection of most of the acid-producing stomach from the esophagus as discussed above, regurgitation from the gastric pouch can persist, and remain refractory to medical management. MSA implantation has been described after RYGB with promising results.^33^ However, further investigation is warranted to better understand the role and outcomes of MSA implantation in the setting of refractory GERD after RYGB.

A primary management option for achalasia spectrum disorders is Heller myotomy, at which time a partial fundoplication is typically performed given decreased esophageal clearance of refluxate in the setting of esophageal motor dysfunction. MSA may have a potential role as an antireflux intervention with Heller myotomy or following per-oral endoscopic myotomy (POEM). However, more data are needed to better understand the risks of dysphagia with MSA implantation representing an obstruction in the setting of esophageal aperistalsis typical of achalasia, as well as any potential increase in risk of MSA device erosion in the setting of prior myotomy.

Although rigorous outcome data are lacking, there has also been interest in expanding indications for MSA to patients with weakly acidic reflux. Among 67 patients with preoperative weakly acidic reflux detected on pH-impedance monitoring, with median follow-up of 24 months, GERD-HRQL improved from 20 to 2, with symptom improvement of 95% for heartburn and regurgitation, similar to outcomes noted in those with GERD.^34^ However, further data are needed regarding the efficacy of MSA for this population before this can be recommended.

Patients with Barrett’s esophagus tend to have significant reflux and could have less esophageal perception of reflux compared with patients without Barrett’s esophagus.^9^ Therefore, MSA could be an option in these patients, especially following endoscopic intervention for dysplasia, where control of esophageal reflux exposure is paramount to preventing recurrence. However, conclusive data are lacking, and esophageal hypomotility that could coexist with Barrett’s esophagus will need to be factored into clinical decision-making.

## MSA AND CONCOMITANT HERNIA REPAIR

While the initial data for MSA were acquired in patients without significant EGJ disruption (i.e., hiatus hernias less than 2–3 cm in size), there may be a role for MSA with concomitant hernia repair in the setting of larger hernias. One prospective study of 200 patients with hiatus hernias >3 cm in size treated with MSA at the time of hernia repair reported complete independence from PPI in 94% at a median follow-up of 9 months, with improvement in median GERD-HRQL from 26 to 2.^35^

The potential utility of this combined intervention has also been demonstrated with longer-term outcome data. In one cohort of 192 total patients with MSA with median follow-up of 20 months, mean GERD-HRQL improved from 19 to 5; a subset of 52 patients with larger hernias (≥3 cm in size) who underwent combined MSA and hiatus hernia repair demonstrated a more profound improvement in mean GERD-HRQL of 21 to 4, and <10% required post-MSA PPI therapy.^36^ In 79 patients with combined MSA and hiatal hernia repair with median follow-up of 3 years, median GERD-HRQL improved from 21 to 2, with evidence of hiatus hernia recurrence in 6%.^37^ Consequently, hiatus hernia repair could be combined with MSA as an alternative to fundoplication, although risks and benefits need further research, and this option needs to be offered taking unique aspects of each individual patient into consideration.

## SAFETY AND COMPLICATIONS

The primary mechanism by which MSA prevents reflux is by augmenting EGJ barrier function and by keeping the LES/EGJ closed, thereby preventing retrograde flow of gastric content. The closing force from the magnetized titanium beads can be overcome by intact esophageal peristalsis propelling an ingested bolus through. However, if the closing force is higher than what can be easily overcome by esophageal peristalsis, bolus retention can occur, and the patient presents with post-MSA dysphagia. The same consequence can be expected if there are alternate structural or motor etiologies for dysphagia that are overlooked on the preoperative work-up.

Dysphagia is therefore the most common side effect reported after MSA. Within a cohort of 380 patients, rates of persistent dysphagia >3 months after MSA was 16%.^21^ In another cohort with predominant regurgitation, proportions with dysphagia decreased from 15% to 7% between 6 and 12 months after MSA.^18^ Endoscopic dilation may be considered in this setting, with estimated overall response rates of 67% in this cohort, prior to discussion of device removal. Longer-term follow-up data at 5 years after MSA noted the prevalence of bothersome dysphagia to be 6%, indicating that the dysphagia ultimately improves in the majority of patients.^15^

If dysphagia persists or worsens after MSA despite conservative management and endoscopic dilation, one management option is device removal. Among the first approximately 30,000 devices implanted globally, the 7-year cumulative risk of explantation was estimated at 4.8%, with smaller sizes more likely to be explanted for etiologies that included dysphagia or odynophagia (48%) and persistent GERD (21%).^20^ The 7-year cumulative risk of device erosion was only 0.3%. A single-center series of five patients who underwent MSA explantation with fundoplication reported four of the five patients were off PPI medications at median follow-up of 41 months.^38^ Finally, the potential longer-horizon consequences of intrathoracic titanium implantation and exposure may warrant further monitoring and evaluation as longer-term data become available.

## LONGER-TERM OUTCOMES

Promising longer-term outcomes over follow up of 5 years and longer are now available for MSA. Follow-up of 33 patients 5 years after MSA demonstrated mean GERD-HRQoL score improvement from 26 to 3, with complete PPI discontinuation in 88% of these patients.^17^ In a cohort of 85 patients followed for >5 years, GERD-HRQoL score was 4, and only 15% used PPIs at 5 years, with all reporting the ability to belch or vomit if needed.^15^ Among 124 patients with follow-up of 6–12 years after MSA, mean GERD-HRQoL improved from 20 to 4, with PPI independence in 79% of patients.^39^ In this cohort, predictors of a favorable outcome included age < 40 years and preoperative GERD-HRQoL >15. Based on a retrospective analysis of 170 patients who underwent MSA, potential factors that may predict suboptimal outcomes with MSA include higher BMI, structurally defective sphincter (hiatus hernia), and elevated LES residual pressures.^40^ Short-term symptomatic outcomes predicted longer-term patient satisfaction, based on a retrospective analysis of 98 patients with median follow-up of 46 months after MSA.^41^

## OUTCOMES COMPARED WITH FUNDOPLICATION

Studies comparing MSA to fundoplication generally demonstrate at least equivalent outcomes.^42^ Matched-pair retrospective analysis of 1-year outcomes for 100 patients found similar GERD-HRQoL scores, PPI use, and postoperative dysphagia for MSA and laparoscopic Nissen fundoplication, but with less severe gas-bloat and preserved ability to belch and vomit in the MSA group.^43^ Another propensity-matched study with 1-year outcomes found similar GERD-HRQoL scores and gas-bloat favoring MSA, but suggested higher rates of mild dysphagia and resumption of daily PPI use in the MSA group.^44^ A meta-analysis of three studies comparing MSA to laparoscopic Nissen fundoplication found similar rates of PPI discontinuation, postoperative dysphagia, and gas-bloat, but superior ability to belch (95% vs. 66%) and vomit (94% vs. 50%) after MSA compared with laparoscopic Nissen fundoplication.^45^ Another meta-analysis that included 6 comparative and 13 single-cohort studies comparing MSA to fundoplication found similar rates of postoperative PPI therapy, GERD-HRQoL scores, and dysphagia for MSA and fundoplication, but demonstrated greater ability to belch and less gas-bloating for MSA compared with fundoplication.^46^ A recent multicenter observational registry study (*n* = 631) comparing MSA and fundoplication over 3 years demonstrated similar improvements in GERD-HRQoL scores, PPI discontinuation, and satisfaction, but higher proportions in the MSA group were able to vomit as needed (91% vs. 68%).^47^

While we are not aware of any published direct or randomized comparisons between MSA and transoral incisionless fundoplication, a meta-analysis including 24 studies with 1942 patients found comparable improvements in GERD-HRQoL scores (78–80%), with greater proportions with improvement in regurgitation (91% vs. 73%) and PPI discontinuation (91% vs. 64%) for MSA compared with transoral incisionless fundoplication.^48^

An economic budget impact model for a hypothetical commercial insurance population of 1 million members over a 1-year time horizon estimated a net cost savings of $111,367 with introduction of MSA compared with medical management or laparoscopic Nissen fundoplication, primarily driven by the avoidance of hospital admission.^49^ Further, a prospective observational study comparing MSA and laparoscopic Nissen fundoplication found that at 12 months after surgery, reimbursement for medical expenses decreased by 11% for MSA and 1% for laparoscopic Nissen fundoplication compared with preoperative reimbursement.^50^

Overall, outcomes appear comparable between MSA and other invasive antireflux interventions, with preserved ability to belch or vomit, and lesser gas-bloat compared with laparoscopic fundoplication.

## CONCLUSIONS

When the optimal patient is carefully identified (proven GERD, no dysphagia or esophageal dysmotility, regurgitation-predominant symptoms), MSA represents a valid management option in the GERD treatment pathway and may have benefits over traditional laparoscopic fundoplication. While significant hiatus herniation previously represented an exclusion criterion for MSA, there is accumulating evidence supporting a role for combined MSA and hiatus hernia repair for patients with EGJ disruption. Emerging data also show increased adoption and uptake of MSA, suggesting equivalency or even benefit in terms of healthcare costs compared with alternate GERD management options. While further high-quality data around long-term outcomes and safety, comparisons with other antireflux interventions, and predictors of clinical success are warranted, MSA represents a valuable option in the GERD management algorithm.
